# Cannabis for sleep in older adults: use has outpaced the science

**DOI:** 10.1186/s42238-026-00515-8

**Published:** 2026-10-09

**Authors:** Khaled Draghmeh, Prakash Gupta, Henry Yaggi, Deepak Cyril D’Souza

**Affiliations:** 1 Department of Psychiatry, Yale University School of Medicine, New Haven, United States; 2 VA CT Health Care System, West Haven, United States; 3 The Clinical Neurosciences Research Unit, Connecticut Mental Health Center, New Haven, United States; 4 Department of Internal Medicine, Section of Pulmonary, Critical Care, and Sleep Medicine, Yale University School of Medicine, New Haven, United States

**Keywords:** Cannabis, Cannabinoids, Delta-9-tetrahydrocannabinol, Sleep, CB1 receptors

## Abstract

Cannabis use among older adults, defined here as those over 50 years of age, has increased rapidly, and sleep disturbance is among the most common reasons for use. Yet, despite growing use of cannabis and individual cannabinoids to facilitate sleep, evidence supporting their efficacy, tolerability and safety in older adults remains limited. Most experimental studies of cannabinoids and sleep have been conducted in younger adults, often using products and formulations that differ substantially from those currently available. The subjective perception of improved sleep, which may drive increased use is often not supported by objective polysomnography or actigraphy data. Aging is accompanied by changes in sleep physiology, pharmacokinetics, body composition, the endocannabinoid system, multimorbidity, and polypharmacy that may alter cannabinoid effects. As a result, findings derived from younger populations may not generalize to older adults. Furthermore, regular and repeated use of tetrahydrocannabinol (THC) and cannabis leads to the development of tolerance and withdrawal-related sleep disruption complicates interpretation of longer-term effects. The evidence for cannabidiol (CBD) and cannabinol (CBN) in sleep remains sparse. Rigorous studies specifically enrolling older adults are virtually nonexistent. Given the rapid growth of cannabis use for sleep in this population, randomized controlled trials incorporating both subjective and objective sleep measures, age-specific pharmacokinetics, drug interactions, and longer-term outcomes are urgently needed to guide clinical care.

## Introduction

Cannabis use among older adults has increased dramatically over the past two decades, making adults aged 65 years and older one of the fastest-growing groups of cannabis users. (Han et al. 2025) The 2024 National Survey on Drug Use and Health (NSDUH) estimated that past-year cannabis use reached 15.0% among all persons over the age of 50 years and 10.5% among adults 65 years of age and older (SAMHSA 2025). Notably, older women constitute one of the fastest-growing populations of cannabis users, and the gap in the prevalence of cannabis use between the two sexes has been decreasing (Chapman et al. 2017; Esie et al. 2026). Although there is a lack of consensus on how younger and older adults are defined in the cannabis literature, in this commentary we define older adults as 50 years and older, consistent with the National Institute on Drug Abuse and other recent studies. (Han et al. 2025; SAMHSA 2025); D’Souza et al. 2026; Pearlson et al. 2026)

Sleep disturbance is among the most common reasons older adults report using cannabis and individual cannabinoids (Yang et al. 2021), reflecting both the high prevalence of insomnia in later life and growing interest in alternatives to sedative hypnotics. As legalization has expanded and commercial products have become increasingly available, many older adults are using tetrahydrocannabinol (THC)- and cannabidiol (CBD)-containing products despite limited scientific evidence supporting their efficacy and safety for insomnia.

This trend has created an important clinical paradox. Although cannabis is increasingly used as a sleep aid by older adults, nearly all evidence on its effects on sleep is derived from studies conducted in younger adults using products that differ substantially from those currently available. This applies to both experimental studies (e.g., double-blind, placebo-controlled laboratory studies of cannabinoid administration) and observational studies (e.g., cross-sectional surveys and cohort studies linking self-reported use to sleep outcomes). In general, the dearth of studies examining the effects of cannabis or cannabinoids in older adults—including the limited studies of pharmacokinetics, acute effects, cognition, and driving—underscores the need for further research in this population. (Ahmed et al. 2014; D’Souza et al. 2026; Di Ciano et al. 2024; Mueller et al. 2021; Pearlson et al. 2026)

We summarize how aging is accompanied by changes in sleep physiology, pharmacokinetics, body composition, the endocannabinoid system, multimorbidity, and polypharmacy, all of which may alter both the therapeutic and adverse effects of cannabinoids (Bilkei-Gorzo 2012; Brown and Winterstein 2019; Velzeboer et al. 2022). We discuss why aging may fundamentally alter cannabinoid responses, review the current evidence regarding cannabinoids and sleep, and identify the research priorities needed to guide evidence-based clinical care.

### Cannabis use for sleep in older adults is rising faster than the evidence to guide It

Cannabis use among older adults has increased substantially over the past two decades, with older adults representing one of the fastest-growing populations of cannabis users (Han et al. 2025). Unlike younger adults, who more commonly report recreational use, older adults frequently use cannabis to manage symptoms (Yang et al. 2021). Sleep disturbance consistently ranks among the most commonly reported indications for use in this population (Yang et al. 2021). This trend is unsurprising given that insomnia affects up to half of older adults and is associated with impaired quality of life, cognitive decline, falls, cardiovascular disease, and depression (Foley et al. 2004; Miner and Kryger 2017). They often view cannabis as a “natural” and safer alternative to prescription sedative hypnotics (Sexton et al. 2016).

Products commonly sold directly to patients through dispensaries, retail stores, or online are not approved by the U.S. Food and Drug Administration and often make claims that are not supported by adequate scientific evidence. Moreover, these products vary considerably in cannabinoid composition, potency, formulation, and route of administration, making it difficult to extrapolate findings from controlled laboratory studies to products currently used in the community.

The mismatch between widespread use and limited scientific evidence is striking. Most studies evaluating cannabinoids and sleep have been conducted in young adults using products that differ from those currently available, while rigorously controlled studies in older adults are virtually nonexistent. Consequently, clinicians are increasingly required to counsel patients based on information derived largely from younger populations whose physiology, comorbidities, and medication use differ substantially from those of older adults.

### Why older adults should not be considered older versions of younger cannabis users

Aging is accompanied by changes in body composition, hepatic and renal function, and other physiological processes that can alter the pharmacokinetics and pharmacodynamics of many drugs.(Mangoni and Jackson 2004). These changes could also influence cannabinoid exposure and effects; however, remarkably little is known about whether the pharmacokinetics of THC, CBD, or CBN differ between younger and older adults. (PA Costa and De Aquino 2026) Small studies of oral THC in older adults demonstrate substantial interindividual pharmacokinetic variability but do not establish an independent effect of age. This leaves clinicians with little evidence to guide dosing or predict adverse effects. (Page et al. 2020) Older adults also disproportionately use oral formulations, such as edibles, oils, and capsules, (Hawkins et al. 2026; Hsu et al. 2026; Tumati et al. 2022) which have a slow onset of effects, greater interindividual variability, and longer duration of action than inhaled cannabis (Grotenhermen 2003). This leaves clinicians with little evidence to guide dosing or predict adverse effects. (Page et al. 2020) Consequently, clinicians are increasingly asked to counsel older patients about cannabis and cannabinoid use despite having little direct evidence to guide such recommendations. (Kansagara et al. 2026; Rubin 2017; Syed et al. 2025; Tian et al. 2026) Available evidence derives largely from younger populations whose physiology, comorbidities, medication use, and patterns of cannabis exposure may differ substantially from those of older adults.

Second, older adults are more likely to have multiple chronic medical conditions (Salive 2013), and as a result, take multiple medications.(Innes et al. 2024; Pan et al. 2024; Tian et al. 2026) Cannabinoids, particularly CBD, have the potential to interact with commonly prescribed medications through cytochrome P450-mediated mechanisms (Brown and Winterstein 2019), raising concerns regarding altered drug exposure, excessive sedation, and other clinically significant adverse events (Brown and Winterstein 2019). Despite the frequency of polypharmacy in older adults, clinically relevant cannabinoid-drug interactions remain poorly characterized. In addition to pharmacokinetic interactions, cannabinoids may have pharmacodynamic interactions with other prescription drugs (e.g., benzodiazepines) and alcohol. The concern for drug-drug interactions is the highest in older adults, who are more likely to use oral cannabis which undergoes first pass metabolism through the liver. (PA Costa and De Aquino 2026; Smith and Gruber 2022) Inhaled cannabinoids bypass initial first-pass metabolism, although clinically relevant pharmacokinetic and pharmacodynamic interactions may still occur.

Third, aging is associated with alterations in the endocannabinoid system itself. Preclinical studies and emerging human imaging data suggest age-related reductions in CB1 receptor availability and changes in endocannabinoid signaling (Bilkei-Gorzo 2012; Radhakrishnan et al. 2022). Because the endocannabinoid system plays an important role in sleep regulation and is the principal biological target of THC, these changes may fundamentally alter cannabinoid responses later in life. Whether they enhance, diminish, or otherwise modify therapeutic effects remains unknown.

Finally, important age- and sex-specific knowledge gaps remain. Sleep disturbance is particularly common during the menopausal transition, and increasing numbers of peri- and postmenopausal women report using cannabis to improve sleep (Dahlgren et al. 2022). However, controlled studies evaluating the effects of THC, CBD, CBN, or contemporary cannabis products in this population are virtually nonexistent. Given known age-relevant sex differences in cannabinoid pharmacology and the influence of reproductive hormones on both sleep and the endocannabinoid system, menopause represents an important but understudied clinical context (Manson and Kaunitz 2016).

Collectively, these biological and clinical considerations suggest that older adults should not simply be viewed as older versions of younger cannabis users. Rather, they represent a distinct population in whom cannabinoid efficacy, tolerability, optimal dosing, and safety may differ substantially. This distinction provides the biological rationale for studying cannabinoids specifically in older adults.

### What do we actually know about cannabinoids and sleep?

Despite the widespread perception that cannabis improves sleep (Babson et al. 2017), the evidence supporting its use remains limited and is derived extensively from studies with sparse representation of older adults.(Mao et al. 2025) Most experimental investigations have evaluated THC under controlled laboratory conditions, whereas considerably less is known about CBD, CBN, or the diverse cannabinoid formulations currently available to consumers (Suraev et al. 2020). Consequently, the existing literature provides only a partial foundation for understanding the effects of cannabis and its constituents on sleep in older adults.

People who use cannabis to facilitate sleep often report improvement in self-reported measures of sleep quality. (da Silva et al. 2025). However, perceived sleep improvement does not necessarily correspond to measurable changes in sleep physiology. Discrepancies between subjective and objective measurements of sleep are well-recognized in insomnia research, with patients sometimes reporting substantial changes in sleep quality or duration despite relatively modest or no changes on polysomnography (PSG) or actigraphy. (Rezaie et al. 2018) Recent systematic reviews incorporating objective sleep measures do not demonstrate consistent effects of cannabis or cannabinoids on sleep duration, latency, wake time, efficiency, or sleep stages. Suppression of REM sleep, while reported more consistently in early trials, has been less consistent in recent trials using doses within the therapeutic range (2.5 mg- 30 mg).(Velzeboer et al. 2022, 2025; Webber et al. 2025).

Acute administration of THC has been associated with modest changes in some sleep parameters, including reduced sleep onset latency, increased total sleep time, and, in some studies, increased slow-wave sleep and reduced rapid eye movement (REM) sleep (Babson et al. 2017; Suraev et al. 2020). Suppression of REM sleep, while reported consistently in early trials, is reported less consistently in recent trials that use doses within the therapeutic range. In general, these effects have not been observed consistently, since many older studies were limited by small sample sizes, heterogeneous methodologies, and short treatment duration. (Velzeboer et al. 2025) Most studies that were conducted decades ago used cannabinoid preparations that differ substantially from products currently available in legal markets.

The effects of repeated cannabinoid exposure are different from those observed following acute administration. Tolerance develops to many of the behavioral and physiological effects of THC (Jones 1983). As tolerance develops, initial improvements in sleep may diminish, and chronic use may be associated with disruption of normal sleep architecture (Bolla et al. 2008). This may prompt some individuals to increase the amount or frequency of cannabis use in an attempt to restore its initial benefits. This, in turn, raises concerns regarding long-term cannabis use and the development of cannabis dependence and cannabis use disorder.

Cannabis exposure history may further modify cannabinoid responses in older adults. Older users include late-life initiators, long-term or continuous users, and individuals who used cannabis earlier in life and resumed after prolonged abstinence (Dahlke et al. 2024; Lloyd and Striley 2018). Chronic frequent THC exposure produces tolerance and CB1 receptor downregulation, which reverses substantially with abstinence (D’Souza et al. 2016). Thus, frequency, intensity, and recency of cannabis exposure, not simply chronological age or lifetime use, may importantly influence cannabinoid effects in older adults.

If individuals who use cannabis regularly reduce or stop using, they may experience a withdrawal syndrome, a hallmark feature of which is sleep disturbance (Budney et al. 2003). Both subjective reports and polysomnographic studies demonstrate that abrupt discontinuation of cannabis is associated with difficulty initiating and maintaining sleep, reduced sleep efficiency, and alterations in sleep architecture (Bolla et al. 2010). Importantly, withdrawal-related insomnia is among the most common reasons individuals resume cannabis use after attempting to stop, suggesting that improvements in sleep reported by some chronic users may, in part, reflect relief of withdrawal symptoms rather than sustained therapeutic benefit (Allsop et al. 2014).

In addition to THC, CBD and CBN have been evaluated as sleep-promoting agents, but evidence remains preliminary and highlights potential discrepancies between subjective and objective outcomes. In a placebo-controlled trial of primary insomnia, 150 mg CBD nightly did not improve most subjective sleep measures despite a modest improvement in objectively measured sleep efficiency. (Narayan et al. 2024) Other trials of CBD and CBN have reported improvements primarily in self-reported sleep disturbance or nighttime awakenings, although findings have been inconsistent and adding CBD to CBN has not clearly enhanced efficacy (Bonn-Miller et al. 2024; Kolobaric et al. 2024; Narayan et al. 2025; Saleska et al. 2024).These findings underscore the importance of assessing both perceived sleep benefit and objective sleep using actigraphy or polysomnography. Moreover, the relatively high doses of purified CBD and CBN studied in clinical trials may differ substantially from amounts present in whole-plant cannabis; thus; findings from purified cannabinoids therefore should not be assumed to apply to cannabis itself. Although these cannabinoids are widely marketed for insomnia in non–FDA-approved contexts, doses and formulations may vary substantially, and many marketed products contain multiple cannabinoids, making it difficult to determine the contribution of individual constituents. At present, there is insufficient evidence to conclude whether CBD, CBN, or combined cannabinoid formulations improve sleep quality, alter sleep architecture, or provide durable clinical benefit.

Perhaps the most important limitation of the existing literature is that it is based almost entirely on younger adults. Few studies have specifically enrolled older adults, and virtually none have examined how age-related changes in pharmacology, multimorbidity, polypharmacy, or the discrepancy between subjective and objective measures of cannabinoid effects on sleep. Consequently, the principal evidence informing real-world use is derived from populations that likely differ substantially from those most likely to use cannabis for insomnia. This disconnect represents an important and underappreciated gap in the cannabinoid literature.

### Clinical implications and research priorities

The growing use of cannabis for sleep among older adults places clinicians in a difficult position. Patients increasingly seek advice regarding THC-, CBD-, and CBN-containing products, yet (Page et al. 2020) clinicians have little direct evidence to guide recommendations for this population. Until adequately powered randomized controlled trials become available, evidence-based treatments for chronic insomnia should remain the first-line therapy. At the same time, clinicians should routinely inquire about cannabis and cannabinoid use when evaluating older adults with sleep complaints, as many patients may not volunteer this information unless specifically asked.

Importantly, the absence of evidence should not be interpreted as evidence of either benefit or harm. Existing studies suggest that cannabinoids may exert modest short-term effects on sleep, but their long-term efficacy and safety in older adults remain largely unknown. Clinicians should therefore discuss the uncertainty surrounding cannabinoid composition of cannabis, dosing, tolerance, withdrawal, potential drug-drug interactions, cognitive impairment, falls, cardiovascular effects, and driving safety when counseling older adults who are considering or already using cannabis or cannabis derivatives for insomnia.

Several research priorities emerge from the current evidence gap. First, adequately powered randomized, double-blind, placebo-controlled trials specifically enrolling older adults are urgently needed. These studies should evaluate THC, CBD, CBN, and commonly used cannabinoid combinations using formulations that reflect products currently available to consumers. Second, studies should incorporate both subjective and objective assessments of sleep. To date, much of the cannabinoid literature has relied on self-reported sleep quality, despite well-recognized discrepancies between subjective sleep measures and objective measures such as polysomnography and actigraphy. Future trials should therefore include objective assessments of sleep architecture, sleep continuity, and circadian timing in addition to validated patient-reported outcomes. Third, studies should define age-specific pharmacokinetics, optimal dosing, and clinically important pharmacokinetic and pharmacodynamic drug interactions. Finally, longitudinal studies are needed to determine whether tolerance, withdrawal, dependence, and cannabis use disorder develop differently in older adults than in younger populations.

## Conclusions

Cannabis use for sleep among older adults is increasing far more rapidly than the scientific evidence supporting its use. Although findings from studies in younger adults provide an important foundation, they cannot replace the need for high-quality studies older populations whose sleep physiology, pharmacology, multimorbidity, and patterns of medication use differ substantially. Older adults represent a biologically and clinically distinct population in whom both the therapeutic effects and risks of cannabinoids may differ meaningfully from those observed in younger individuals.

Closing this evidence gap should become a research priority. Rigorous studies conducted specifically in older adults are needed to determine whether cannabinoids improve sleep, identify those most likely to benefit or be harmed, and provide the evidence necessary for clinicians, patients, and policymakers to make informed decisions regarding the use of cannabis for insomnia in later life.

## Data Availability

No datasets were generated or analysed during the current study.
